# Effects of chirality on the intracellular localization of binuclear ruthenium(II) polypyridyl complexes

**DOI:** 10.1007/s00775-012-0877-0

**Published:** 2012-02-05

**Authors:** Frida R. Svensson, Johanna Andersson, Helene L. Åmand, Per Lincoln

**Affiliations:** Department of Chemical and Biological Engineering, Chalmers University of Technology, Kemivägen 10, 41296 Gothenburg, Sweden

**Keywords:** Cellular uptake, DNA, Enantioselectivity, Imaging agents, Ruthenium

## Abstract

Interest in binuclear ruthenium(II) polypyridyl complexes as luminescent cellular imaging agents and for biomedical applications is increasing rapidly. We have investigated the cellular localization, uptake, and biomolecular interactions of the pure enantiomers of two structural isomers of [μ-bipb(phen)_4_Ru_2_]^4+^ (bipb is bis(imidazo[4,5-*f*]-1,10-phenanthrolin-2-yl)benzene and phen is 1,10-phenanthroline) using confocal laser scanning microscopy, emission spectroscopy, and linear dichroism. Both complexes display distinct enantiomeric differences in the staining pattern of fixed cells, which are concluded to arise from chiral discrimination in the binding to intracellular components. Uptake of complexes in live cells is efficient and nontoxic at 5 μM, and occurs through an energy-dependent mechanism. No differences in uptake are observed between the structural isomers or the enantiomers, suggesting that the interactions triggering uptake are rather insensitive to structural variations. Altogether, these findings show that the complexes investigated are promising for future applications as cellular imaging probes. In addition, linear dichroism shows that the complexes exhibit DNA-condensing properties, making them interesting as potential gene delivery vectors.

**Electronic supplementary material:**

The online version of this article (doi:10.1007/s00775-012-0877-0) contains supplementary material, which is available to authorized users.

## Introduction

Heavy-metal coordination complexes have recently emerged as a novel class of biological imaging agents for microscopy applications [1, 2]. Among these, ruthenium(II) polypyridyl complexes have attracted increasing interest as probes that selectively stain particular cellular compartments [3] or certain biomolecules [4–6], or monitor cell viability [7, 8]. The advantages of using such complexes as cellular staining agents, compared with conventional organic fluorescent dyes, include large Stokes shifts, high photostability, red emission wavelengths, and long and environmentally sensitive excited-state lifetimes. Additionally, the photophysical properties can be modified by systematically varying the ligands, and the octahedral symmetry of ruthenium(II) polypyridyl complexes also enables synthesis of stable enantiomers, Δ and Λ, that may probe chiral environments [9, 10].

Ruthenium polypyridyl complexes have been extensively studied during the last three decades for their strong and sequence-selective DNA binding [5, 11, 12]. Attempts to further improve DNA affinity and target more specific structures have resulted in an increasing interest in binuclear complexes, and to date, there are several examples of binuclear ruthenium complexes showing potential for therapeutic use. The preferential binding of certain binuclear ruthenium complexes to either AT-rich sequences [13–15] or structural DNA and RNA features such as bulges [16–18] or telomere quadruplexes [5, 19] could possibly enable targeting of the AT-rich malaria parasite genome, bulge sites in HIV-1 sequences, or cancer cells having enhanced telomerase activity, respectively. There are also examples in the literature of binuclear ruthenium complexes that condense DNA and thus could have the ability to function as gene delivery vectors or to control gene expression [13].

Despite the attractive photophysical properties and promising DNA-binding characteristics of binuclear ruthenium complexes, there have been relatively few studies focusing on the interaction between these complexes and live cells [3, 4, 20, 21]. As a consequence, knowledge regarding cellular uptake, intracellular localization, biomolecular binding, and the influence of enantiomeric differences for these events is limited. These important questions thus need to be addressed before successful use of ruthenium complexes for biomedical applications and as cellular imaging probes is possible.

**Scheme 1:**
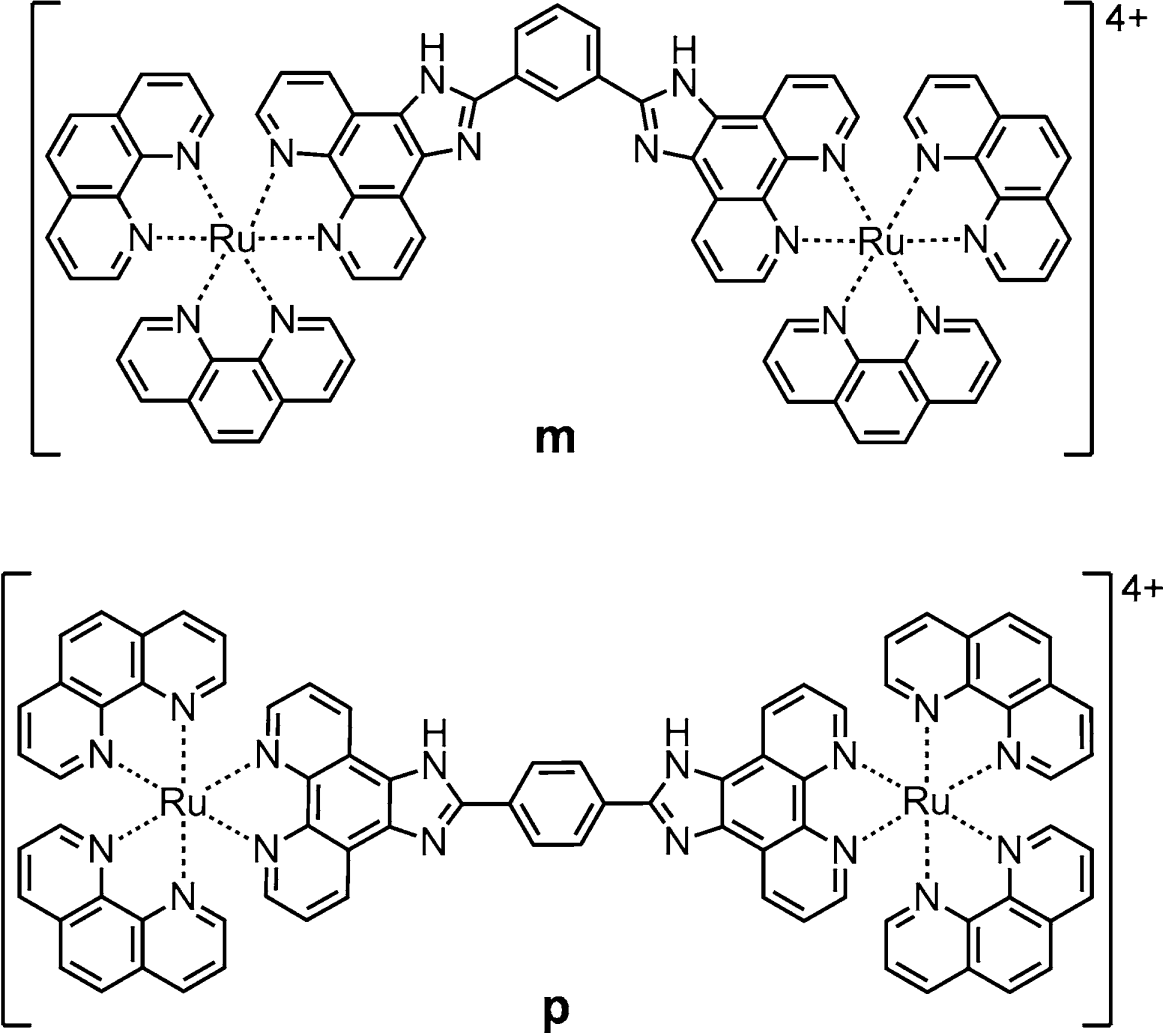
Structures of the ruthenium complexes [μ-*meta*-bipb(phen)_4_Ru_2_]^4+^ (**m**) and [μ-*para*-bipb(phen)_4_Ru_2_]^4+^ (**p**), where *meta*-bipb is 1,3-bis(imidazo[4,5-*f*]-1,10-phenanthrolin-2-yl)benzene and *para*-bipb is 1,4-bis(imidazo[4,5-*f*]-1,10-phenanthrolin-2-yl)benzene

In this work, we investigated the interactions between the enantiomerically pure forms, ΔΔ and ΛΛ, of two structural isomers of a binuclear ruthenium complex, the previously reported [μ-*meta*-bipb(phen)_4_Ru_2_]^4+^ (denoted **m**; *meta*-bipb is 1,3-bis(imidazo[4,5-*f*]-1,10-phenanthrolin-2-yl)benzene and phen is 1,10-phenanthroline) [13, 22] and the new complex [μ-*para*-bipb(phen)_4_Ru_2_]^4+^ (denoted **p**; *para*-bipb is 1,4-bis(imidazo[4,5-*f*]-1,10-phenanthrolin-2-yl)benzene), with mammalian CHO-K1 cells, to shed light on the potential of binuclear ruthenium(II) polypyridyl complexes as cellular imaging probes (Scheme 1). The localization in fixed cells and binding to pure bioenvironments are compared for the four complexes to evaluate the effect of both chirality and small structural differences on their affinity for different cellular components. Moreover, the uptake and localization in live cells is studied, which is possible since **m** and **p**, in contrast to the well-known dipyridophenazine (dppz) complexes [10, 23] have no “light-switch” effect, and hence are emissive in all environments.

## Materials and methods

### Materials

The Chinese hamster ovary (CHO-K1) cell line was a kind gift from Ülo Langel, Stockholm University. Cell culture reagents (Ham’s F-12 medium, fetal bovine serum, trypsin, and l-glutamine) were from PAA Laboratories. The nucleic acid stain probe Sytox^®^ Green (impermeable to live cells) was purchased from Invitrogen. All biophysical experiments were performed in 1 mM cacodylate buffer (pH 7.1) with 150 mM NaCl.

### Synthesis

ΔΔ-**m** and ΛΛ-**m** were synthesized as described elsewhere [13]. ΔΔ-**p** and ΛΛ-**p** were synthesized from homochiral bis(1,10-phenanthroline)(1,10-phenanthroline-5,6-dione)ruthenium bis(hexafluorophosphate), prepared as previously reported by Hiort et al. [10], and terephtalaldehyde (Sigma-Aldrich) according to the same procedure as used for **m**. ^1^H NMR (400 MHz, acetonitrile-*d*_3_): *δ* = 9.13 (d, *J* = 8.0 Hz, 4H), 8.61 (*J* = 8.0 Hz, 8H), 8.55 (s, 4H), 8.26 (s, 8H), 8.10 (d, *J* = 5.3 Hz, 4H), 8.03 (d, *J* = 5.3 Hz, 4H), 8.00 (d, *J* = 6.0 Hz, 4H), 7.60–7.75 (m, 12H). Mass spectrometry (matrix-assisted laser desorption/ionization time of flight, sinapic acid matrix): *m*/*z*: calcd for [M]^+^: 1,438.2, found 1,437.3 [M−H]^+^. For absorption spectra, see Fig. S1.

### Cell culture

CHO-K1 cells were cultured in Ham’s F-12 medium supplemented with fetal bovine serum (10%) and l-glutamine (2 mM) at 37 °C and 5% CO_2_. Two days before the experiment, approximately 80,000 cells were seeded in glass-bottom dishes (WillcoWells, Netherlands). The cells were fixed by addition of methanol at −20 °C for 15 min, and thereafter rinsed once with serum-free medium before incubation with the complex (5 μM diluted in serum-free medium) for 15 min. The cells were rinsed once with the medium before imaging. For live-cell imaging, cells were rinsed and incubated with the complex for 1 h at 37 °C before imaging unless otherwise stated. For cellular uptake experiments where endocytosis was inhibited, cells were incubated with the complex for 1 h at 4 °C before imaging.

### Confocal microscopy

Images were acquired using an HCX PL APO ×63/1.32 oil immersion objective on a Leica TCS SP2 RS confocal microscope (Wetzlar, Germany). The 488-nm line of the argon laser was used for excitation of the ruthenium complexes, and emission was detected at 600–700 nm. Sytox Green was also excited at 488 nm, and emission was detected between 500 and 550 nm. The photomultiplier tube voltage and gain were optimized for each image. All experiments were repeated at least twice and representative images are presented in this article.

### Steady-state emission spectroscopy

Steady-state emission measurements were performed with a Cary Eclipse fluorescence spectrophotometer (Varian, USA) at room temperature. The excitation wavelength was 460 nm and the emission was measured between 500 and 850 nm with excitation and emission slits of 5 nm. Quantum yields were determined by comparing the absorbance-weighted integrated emission intensities using Ru(phen)_2_(11,12-dimethyldipyridophenazine) in 1,2-propanediol as a reference (reported quantum yield of 7.7%) [24].

### Preparation of large unilamellar vesicles

Phospholipid vesicles of 1,2-dioleoyl-*sn*-glycero-3-phosphatidylcholine and 1,2-dioleoyl-*sn*-glycero-3-phosphatidylglycerol at a lipid molar ratio of 4:1 were prepared by the extrusion method. Lipids dissolved in chloroform were mixed in a round-bottom flask. The solvent was evaporated under reduced pressure using a rotary evaporator followed by drying in high vacuum (minimum 2 h) to ensure that remaining traces of chloroform were removed. Vesicles were formed by addition of buffer to the lipid film followed by vortexing. Five freeze–thaw cycles (N_2_(l)/37 °C) and extrusion 21 times through polycarbonate filters of 100-nm pore size using a handheld syringe extruder rendered unilamellar lipid vesicles of a diameter of approximately 100 nm.

### Linear dichroism

Linear dichroism (LD) is defined as the differential absorption of linearly polarized light, parallel and perpendicular to a macroscopic orientation axis:

1 $$ {\text{LD = }}A_{||} - A_{ \bot } . $$

The technique requires an oriented sample, which was obtained here in the shear flow of a rotating Couette cell. A sufficiently long DNA helix aligns in the flow field with the bases on average perpendicular to the orientation axis, which results in a negative LD signal at 260 nm. The magnitude of the LD signal is dependent on the degree of orientation of the sample, and hence molecular interactions that affect the DNA helix, such as condensation, can be investigated by the LD technique [25, 26]. LD was measured with a JASCO J-720 circular dichroism spectropolarimeter equipped with an Oxley prism to obtain linearly polarized light, using a Couette cell with a 1-mm path length. Spectra were measured between 200 and 500 nm. Samples were prepared by mixing 1:1 volumes of calf thymus DNA and the ruthenium complex diluted in buffer.

## Results

### Cellular localization

**Fig. 1:**
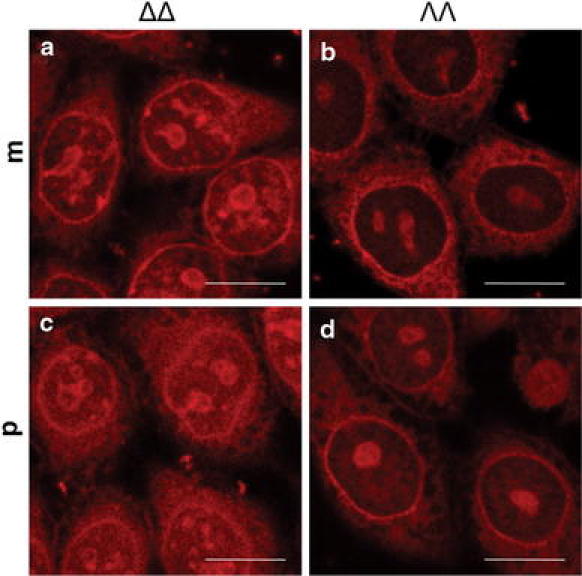
Confocal laser scanning microscopy (CLSM) images of *a* ΔΔ-**m**, *b* ΛΛ-**m**, *c* ΔΔ-**p**, and *d* ΛΛ-**p**, in fixed cells. Cells fixed with methanol were incubated with 5 μM ruthenium complex for 4 h before imaging. *Scale bars* 10 μm

Figure 1 shows confocal laser scanning microscopy (CLSM) images of the intracellular localization of both enantiomers (ΔΔ and ΛΛ) of the two structural isomers (**m** and **p**) in fixed CHO-K1 cells. Interestingly, the staining patterns in fixed cells are similar for **m** and **p**, but there are significant differences between the enantiomers of the two complexes. The ΔΔ enantiomers of both **m** and **p** show more intense and structured emission inside the nucleus (Fig. 1, images a, c) compared with the ΛΛ enantiomers. For the ΛΛ enantiomers, the emission in the nucleus is generally lower relative to that in the cytoplasm although pronounced staining of the nucleoli is observed (Fig. 1, images b, d). Intense nuclear membrane staining was observed for all four complexes.

### Biophysical characterization of emission properties

**Fig. 2:**
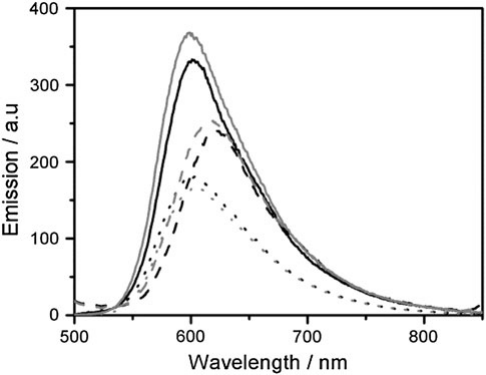
Emission spectra of ΔΔ-**p** (2 μM, *black lines*) and ΛΛ-**p** (2 μM, *gray lines*) in calf thymus DNA (40 μM, *solid lines*), 1,2-dioleoyl-*sn*-glycero-3-phosphatidylcholine/1,2-dioleoyl-*sn*-glycero-3-phosphatidylglycerol large unilamellar vesicles (100 μM, *dashed lines*), and buffer (150 mM NaCl, *dotted lines*). The spectra are normalized against the absorption at 460 nm

To reveal whether the different staining patterns in fixed cells for the ΔΔ and ΛΛ enantiomers are due to differences in affinity, and hence concentration differences in the nucleus, or to variations in photophysical properties when they are bound to the intracellular components, emission spectra of the complexes bound to pure bioenvironments were measured. Figure 2 shows emission spectra of ΔΔ-**p** and ΛΛ-**p** bound to calf thymus DNA, to phospholipid vesicles (large unilamellar vesicles, LUVs), and in buffer. For details of the maximum emission wavelengths and quantum yields for both **p** and **m**, see Table 1. There is not a large difference in the emission quantum yield between the two enantiomers in either of the environments tested, and hence their distinct dissimilar cellular staining patterns cannot be explained by differences in quantum yields when they are bound to certain biomolecules. For the **p** complexes, the quantum yields are highest in calf thymus DNA followed by LUVs, whereas for the **m** complexes the emission intensities in these two environments are comparable. Both **m** and **p** complexes also show strong emission in buffer, in contrast to light-switch complexes. Overall, slightly higher quantum yields were observed for **m** compared with **p**. This difference is most accentuated in LUVs, where the quantum yield for **m** is almost twice as large as for **p** (see Table 1). As seen in Fig. 2, the emission from **p** is redshifted in LUVs compared with when it is bound to DNA, which is not observed for **m**.

**Table 1:** Maximum emission wavelengths and quantum yields for **m** and **p** complexes (see Scheme 1 for the structures)

	*λ* (nm)	*Φ* (%)^a^
DNA^b^		
ΔΔ-**m**	602	13.5
ΛΛ-**m**	606	15.8
ΔΔ-**p**	603	10.7
ΛΛ-**p**	600	12.0
LUVs^c^		
ΔΔ-**m**	603	14.0
ΛΛ-**m**	607	15.3
ΔΔ-**p**	624	8.4
ΛΛ-**p**	618	9.0
Buffer^d^		
**m**	605	7.7
**p**	602	6.8

*LUVs* large unilamellar vesicles

^a^As a reference, Ru(phen)_2_(11,12-dimethyldppz) (dppz is dipyridophenazine and phen is 1,10-phenanthroline) in 1,2-propanediol (7.7%) was used [24]. In buffer, mean values for the two enantiomers are presented for **m** and **p** as they were essentially the same.

^b^Complex (2 μM) in calf thymus DNA (40 μM)

^c^Complex (2 μM) in 1,2-dioleoyl-*sn*-glycero-3-phosphatidylcholine/1,2-dioleoyl-*sn*-glycero-3-phosphatidylglycerol LUVs (100 μM)

^d^Complex (2 μM) in buffer

### Linear dichroism

**Fig. 3:**
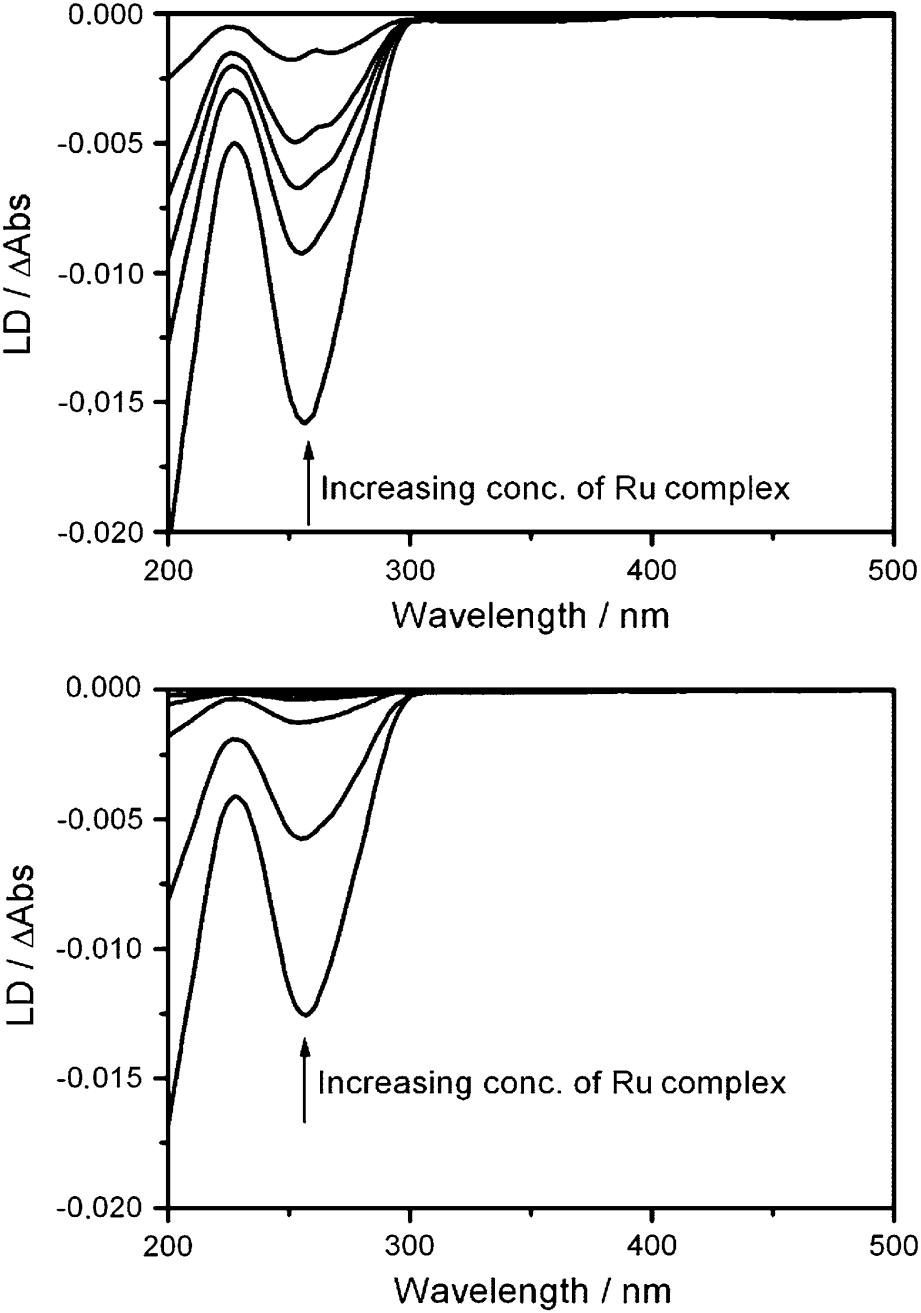
Linear dichroism (*LD*) spectra of calf thymus DNA (100 μM) and after addition of ΔΔ-**p** (*top*) and ΛΛ-**p** (*bottom*) at ruthenium complex concentrations of 0, 1, 2, 3, and 5 μM. The amplitude of the DNA LD signal at 260 nm decreases with increasing ruthenium complex concentration. The concentration of NaCl was 150 mM

Despite the pronounced enantiomeric difference in the staining pattern of fixed cells, the interaction with different bioenvironments appears to be similar for the two enantiomers of both **m** and **p** as judged from emission data. However, for the **m** complex we previously observed enantiomeric differences in interaction with DNA using flow LD, and found that the ΔΔ enantiomer condenses DNA much more efficiently than the ΛΛ enantiomer [13]. To elucidate whether this effect can account for the brighter nuclear staining by the ΔΔ enantiomers, a similar LD study was performed for the **p** complex. Figure 3 shows LD spectra of titrations of the two **p** enantiomers into calf thymus DNA samples of constant concentration. The condensation of DNA increases with increasing complex to base pair ratio, which can be seen as a decrease in the amplitude of the DNA LD signal at 260 nm. This is a consequence of the DNA helix concomitantly losing its orientation in the shear flow of the Couette cell. Interestingly, a pronounced difference between the enantiomers is observed, but contrary to what might be expected, condensation is most efficient for ΛΛ-**p**, where a complete loss of DNA LD signal occurs at a complex to base pair ratio of around 1:16 (Fig. 3b).

### Cellular uptake

**Fig. 4:**
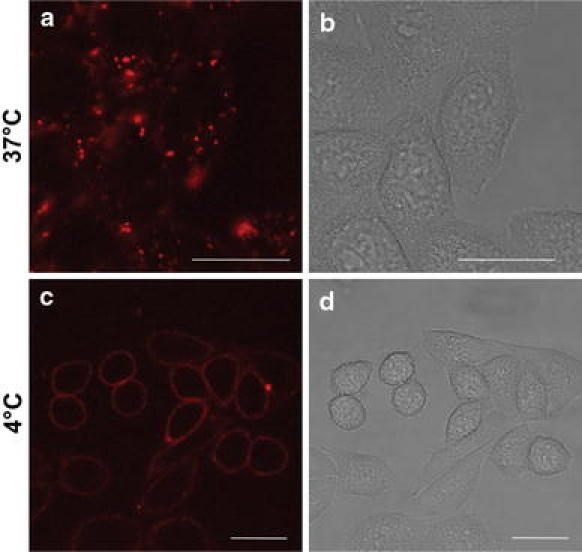
Representative CLSM images of ΔΔ-**m** (5 μM) in live CHO-K1 cells after incubation for 1 h at 37 °C (*a*) and after incubation for 1 h at 4 °C (*c*). *b*, *d* the corresponding transmission images. *Scale bars* 20 μm

Since the two enantiomers of both **m** and **p** show distinctly different staining patterns in fixed cells, we wanted to investigate if chirality influences uptake and intracellular staining also in live cells. Figure 4, image a shows CLSM images of ΔΔ-**m** in live CHO-K1 cells after 1 h incubation with 5 μM complex. ΔΔ-**m** is efficiently internalized even at this low complex concentration, and the punctuate staining pattern indicates uptake via endocytosis. The complex is found solely in the cytoplasm and no nuclear staining is observed. Further evidence that supports uptake via an endocytotic pathway is shown in Fig. 4, image c, where cells kept at 4 °C were incubated with ΔΔ-**m** for 1 h. At this temperature, energy-dependent processes, such as endocytosis, are shut down, and as a result, no cellular uptake is observed. Instead the ruthenium complex remains bound to the plasma membrane. Cellular uptake was also investigated for the other complexes, and no significant differences could be distinguished, neither between **m** and **p** nor between their two enantiomers (see Fig. S2). Despite the efficient cellular uptake, no toxicity was detected at this concentration, as evidenced by retained cell morphology and the absence of staining with the dead-cell marker Sytox Green (Fig. S3).

**Fig. 5:**
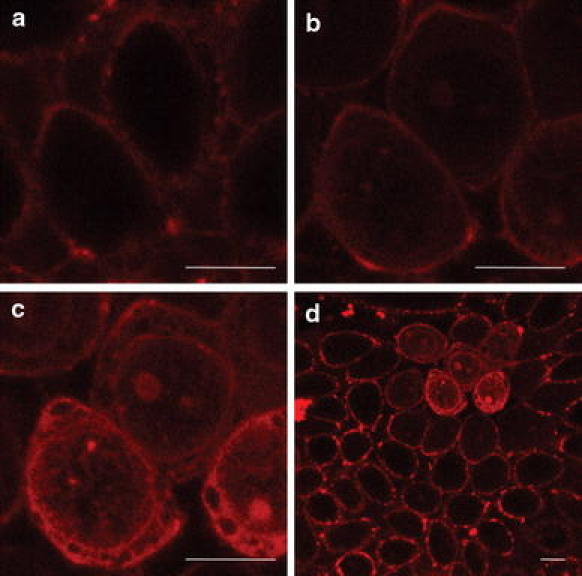
CLSM images of ΛΛ-**p** (5 μM) in live CHO-K1 cells **a** immediately after addition, **b** after 7 min illumination, and **c**, **d** after 13 min illumination with the 488-nm laser. *Scale bars* 10 μm

Uptake of **m** and **p** into live cells can thus be concluded to occur via endocytosis, with concomitant entrapment in endosomes. Although uptake is efficient, lack of endosomal escape can be regarded as a limitation, and therefore we investigated if direct membrane penetration could be achieved through illumination by light. This process is referred to as photoactivated uptake, and has previously been observed for lipophilic mononuclear ruthenium dppz complexes [27, 28]. A few minutes of laser illumination of cells with these complexes extracellularly bound to the membrane causes photodamage to the membrane, resulting in increased permeability and thus accumulation of the complex inside the cells. We found that this phenomenon indeed occurred also for the binuclear **m** and **p** complexes, and Fig. 5 shows ΛΛ-**p**, initially bound to the plasma membrane, being internalized as a result of laser illumination. The final staining pattern resembles that in fixed cells (see Fig. 1, image d). Notably, cells outside the focal point that are not illuminated are unaffected and hence show no uptake of the ruthenium complex on this timescale (Fig. 5d).

## Discussion

Despite the increasing interest in metal–ligand complexes as cellular imaging and DNA-binding agents, little is known regarding their cellular uptake and intracellular biomolecular binding. In this study we explored the effect of small structural variations and chirality of luminescent binuclear ruthenium(II) polypyridyl complexes on their interactions with cells and pure biomimetic environments. The luminescence of these complexes is rather insensitive to the environment and we took advantage of this property to study their intracellular distribution in both fixed and live cells, with the aim to evaluate their potential as cellular imaging probes.

In fixed cells there is a significant enantiomeric difference in the cellular staining pattern for both **m** and **p**, with the ΔΔ enantiomers displaying more prominent nuclear staining (Fig. 1). In contrast to previously studied mononuclear ruthenium complexes, where the brighter staining of the nucleus by the Δ enantiomer compared with the Λ enantiomer was explained by a higher quantum yield for the Δ enantiomer when it is bound to DNA [27], studies of the quantum yield of **m** and **p** in pure bioenvironments reveal very small differences between the enantiomers (Fig. 2, Table 1). Hence, the distinct intracellular staining patterns in fixed cells can only be explained by enantiomeric differences in the affinity for cellular components. The interaction between **p** and DNA was further studied by LD to investigate whether the enantiomeric differences in the staining pattern of fixed cells could be related to more efficient DNA condensation by the ΔΔ enantiomers as previously observed for the **m** complex [13]. As expected, **p** also exhibits a pronounced DNA-condensing capability, but on contrast to what is observed for **m**, the ΛΛ enantiomer is the more efficient condensing agent (Fig. 3). This reversed chirality effect is surprising considering the similar enantiomeric staining patterns in fixed cells, but highlights the complexity of the interactions even in this simple system and shows that the intracellular milieu is chirally discriminating to an extent far beyond pure DNA in solution. The fact that the enantiomeric staining patterns are quite similar for **m** and **p** indicates that chirality is more important than the structure of the complex for the intracellular distribution of these complexes.

The uptake of these binuclear complexes into live cells is efficient and, unlike the interactions with fixed cells, independent of both chirality and structure. Instead, all four complexes are readily internalized by CHO-K1 cells at concentrations as low as 5 μM (Fig. 4). This concentration is 100 times lower than what was used in previously published studies of cellular uptake of binuclear ruthenium complexes [4, 29], and as a comparison, uptake in this micromolar concentration regime is often observed for peptide-based intracellular delivery vectors designed to have a high capacity to enter cells [30–33]. Since the complex is found in dot-like structures and no intracellular staining is observed with incubation at 4 °C, an energy-dependent uptake mechanism, presumably endocytosis, is proposed. This has been observed before for other ruthenium(II) complexes [34, 35], although other mechanisms such as passive diffusion have also been suggested [3, 4, 36]. Entrapment in endosomes is a limitation, but in resemblance to lipophilic mononuclear ruthenium complexes, photoactivated uptake directly through the membrane can be induced by laser illumination of plasma-membrane-bound **m** and **p** complexes [27, 28], which also provides a possible route for selective cellular uptake. Unfortunately, this process results in membrane damage and cell death, and is thus only applicable for imaging applications. To obtain cytoplasmic localization also in live cells, direct membrane penetration could possibly be enhanced by increasing the lipophilicity of these complexes, which has previously been shown for dppz-containing mononuclear ruthenium complexes [28, 36, 37]. Another possible approach is to enhance endosomal escape by the use of endosome-disruptive agents.

The biophysical studies of binding to pure bioenvironments revealed only small differences between the enantiomers. However, when we investigated the ability to condense DNA, both chiral and isomeric effects were observed. The fact that these complexes have a strong capacity to condense DNA, which can be fine-tuned by small structural alterations, also makes them potential candidates for gene delivery. An interest in ruthenium(II) complexes as gene delivery vectors has recently emerged, and successful transfection using mononuclear ruthenium complexes has indeed been reported [38, 39]. In light of these findings, it would be of great interest to investigate the potential of our complexes, displaying great DNA-condensing capability combined with efficient internalization into cells, to function as DNA carriers.

## Conclusion

The results of this study show that binuclear ruthenium(II) polypyridyl complexes possess a number of properties that make them promising as cellular imaging probes. First, the **m** and **p** complexes display distinct enantiomeric differences in intracellular distribution in fixed cells, originating from differential affinity for cellular components. Further, the uptake in live cells is efficient even at low concentrations that are nontoxic to cells. However, whereas the intracellular milieu is highly discriminating with respect to chirality, events such as plasma membrane binding and cellular uptake appear to be insensitive to both enantiomeric effects and structural differences. Possible limitations for binding of these complexes to intracellular targets include accumulation in endosomes, and strategies to circumvent this need to be addressed. Finally, owing to the recent interest in ruthenium(II) complexes as DNA delivery vectors and the observed DNA-condensing capability of the present complexes, it would be of great interest to investigate their potential as DNA carriers.

## Electronic supplementary material

Below is the link to the electronic supplementary material.

Supplementary material 1 (PDF 145 kb)

## Abbreviations

*meta*-bipb: 1,3-Bis(imidazo[4,5-*f*]-1,10-phenanthrolin-2-yl)benzene
*para*-bipb: 1,4-Bis(imidazo[4,5-*f*]-1,10-phenanthrolin-2-yl)benzene
CLSM: Confocal laser scanning microscopy
dppz: Dipyridophenazine
LD: Linear dichroism
LUV: Large unilamellar vesicle
Phen: 1,10-Phenanthroline
