# Supplementary material for: Effects of chirality on the intracellular localization of binuclear ruthenium(II) polypyridyl complexes
Source: J Biol Inorg Chem. 2012 Feb 5;17(4):565–71. doi: 10.1007/s00775-012-0877-0 (PMC3324687; doi:10.1007/s00775-012-0877-0)
Supplement: Supplementary file 1 — Supplementary material 1 (PDF 145 kb) [file 775_2012_877_MOESM1_ESM.pdf]

Supplementary Material

Absorption

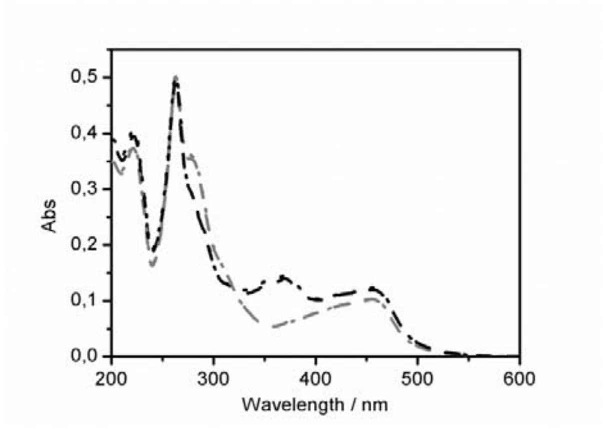

Figure S1. Absorption spectra of ΔΔ-**p** and ΛΛ-**p** (black), and ΔΔ-**m** and ΛΛ-**m** (grey) in 150 mM NaCl buffer. The absorption for the enantiomers are indistinguishable (dashed and dotted lines), and **m** and **p** have similar shapes at 260 nm which is where the π→π* transitions of the phenanthroline ligands absorb. The new π→π* band observed for **p** around 370 nm reflects the efficient π-conjugation of the para-substituted bridging ligand.

Cellular uptake

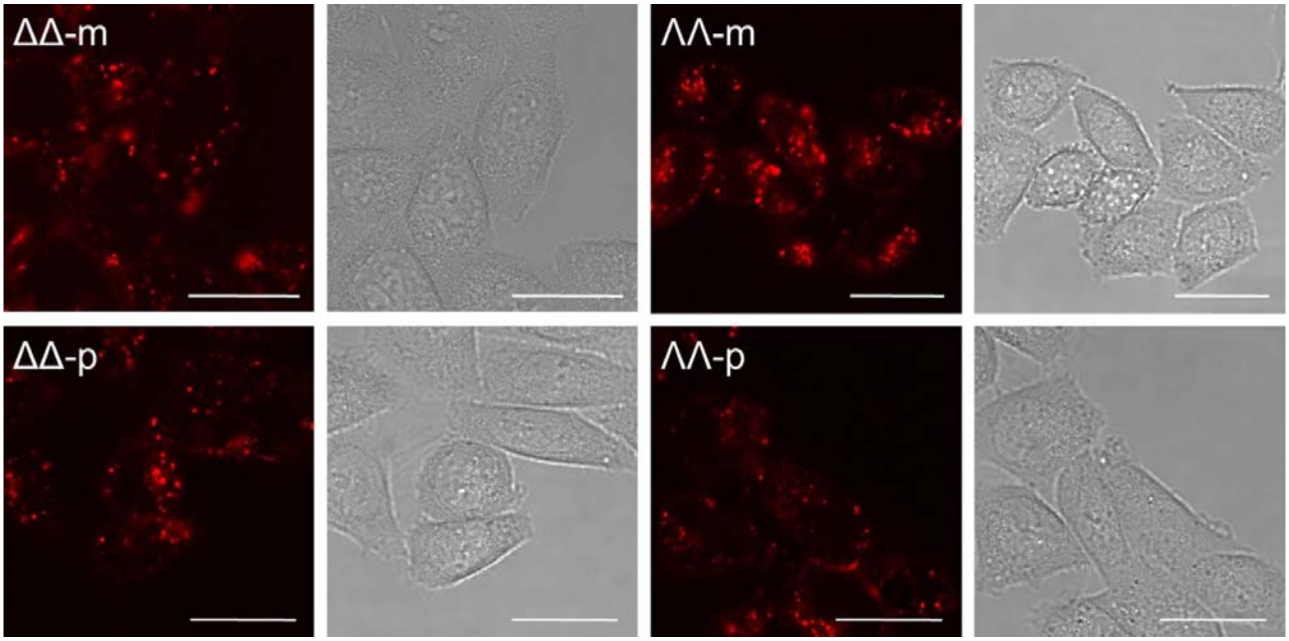

Figure S2. CLSM images of live cells incubated with both enantiomers of **m** and **p** (5 μM, 1 h). Punctuate staining is observed and all four complexes are internalized approximately to the same extent judged by the emission intensity (the PMT and gain was the same for all images). Scale bars are 20 μm.

Cytotoxicity

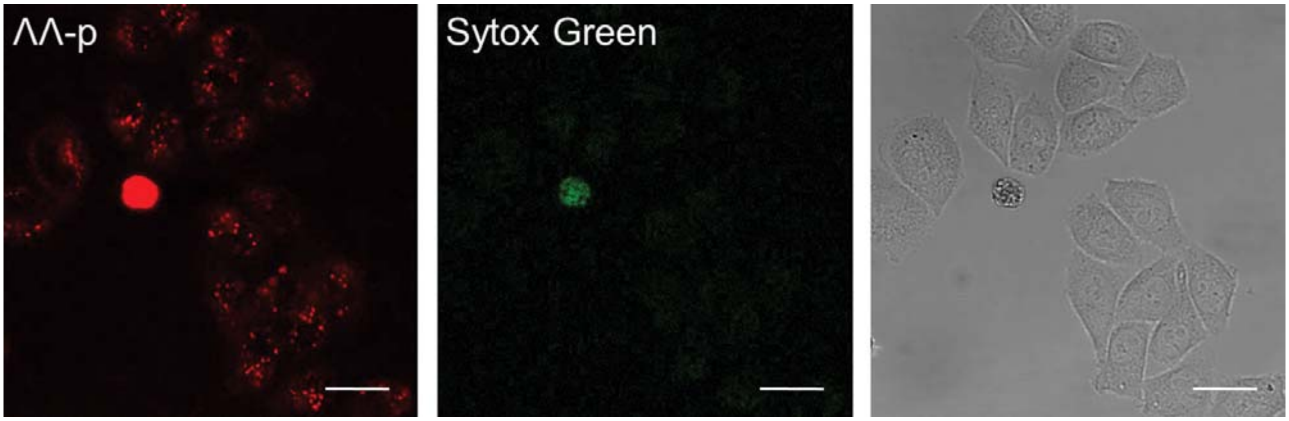

Figure S3. CLSM images of live cells incubated with ΛΛ-**p** (5 µM, 1 h) and the nucleic acid probe Sytox Green (0.1 µM, 10 min). Sytox Green is impermeable to live cells and thus only stains dead cells. Equivalent results are obtained for the ΔΔ enantiomer and for the **m** complexes, which show that these binuclear ruthenium complexes are not cytotoxic after incubation 1 h at low micromolar concentrations.
